# Addition of a McLaughlin cerclage to locking plate fixation of distal patellar pole fractures reduces interfragmentary displacement – a biomechanical human cadaveric study

**DOI:** 10.1007/s00068-026-03380-5

**Published:** 2026-10-27

**Authors:** Adrian Deichsel, Arian Große-Allermann, Klemens Horst, Christoph Kittl, Moritz Lodde, Boyko Gueorguiev, Ludmil Drenchev, Hristo K. Skulev, Elmar Herbst

**Affiliations:** 1 Department of Trauma, Hand and Reconstructive Surgery, University Hospital Münster, Albert- Schweitzer-Campus 1, 48149 Münster, Germany; 2 AO Research Institute Davos, Davos, Switzerland; 3 Institute of Metal Science, Equipment and Technologies with Hydro- and Aerodynamics Centre “Acad. A. Balevski”, Bulgarian Academy of Sciences, Sofia, Bulgaria

**Keywords:** Knee, Fracture, Cerclage, Patella, Biomechanics

## Abstract

**Purpose:**

To investigate whether the addition of a McLaughlin cerclage to locking plate fixation of distal patellar pole fractures provides improved biomechanical construct stability and reduced interfragmentary displacement.

**Methods:**

A distal patellar pole fracture was simulated in sixteen fresh-frozen human cadaveric knees by a horizontal osteotomy in the distal third of the patella. Subsequently, locking plate fixation was performed. In a randomized half of the specimens, an additional McLaughlin wire cerclage was applied. The specimens were then subjected to 3000 cycles of extension starting at 90° of flexion under a maximum tensile load of 320 N. Using a Digital Image Correlation (DIC) system, the interfragmentary displacement between the proximal and distal fragments was measured at 1, 10, 20, 30, 50, 100, 500, 1000, 2000, and 3000 cycles. Statistical analysis was performed using repeated-measures ANOVA and Welch’s unpaired *t*-t test at 3000 cycles.

**Results:**

An additional McLaughlin cerclage significantly reduced interfragmentary displacement under cyclic loading in comparison to locking plate fixation alone. After 3000 cycles, displacement was 2.27 ± 1.84 mm with plate fixation alone and 0.36 ± 0.22 mm with additional McLaughlin cerclage (mean difference 1.91 mm; 95% CI 0.38–3.45 mm; *p* = 0.0215).

**Conclusion:**

An additional McLaughlin cerclage reduced interfragmentary displacement in distal patellar pole fractures. In cases of unstable distal pole patella fractures, the additional use of a McLaughlin cerclage may therefore be considered to enhance construct stability.

## Introduction

Historically, the surgical management of patellar fractures was dominated by tension band wiring (TBW) [24]. However, biomechanical evidence has challenged its proposed mechanism [14, 20, 42]. In clinical data, this results in persistent fracture gaps, implant irritation, and high rates of symptomatic hardware removal [9, 23, 29, 36, 39]. Fixed-angle locking plate osteosynthesis has emerged as a promising alternative, offering superior biomechanical stability [33, 40]. Comparative biomechanical analyses have demonstrated that locked plating withstands significantly higher loads than TBW or lag screw constructs and shows less interfragmentary motion under cyclic loading conditions [2, 16, 31, 34, 35, 37, 41]. However, even modern plate osteosynthesis techniques face challenges when addressing multifragmentary fractures. Fractures involving the distal patellar pole are particularly prone to displacement due to the high tensile forces acting on the patellar tendon [19, 22]. Furthermore, the distal pole is often poorly vascularized, comminuted, and offers limited bone stock, further compromising fixation strength [12, 18, 22].

To augment osteosynthesis of the patella, cerclages can be additionally applied. Originally described for tendon ruptures, a McLaughlin cerclage neutralizes distraction forces by anchoring the patellar tendon to the tibial tuberosity via a horizontal transosseous cerclage wire or suture [1, 3, 4, 21], which might enhance construct stability, possibly reducing failure of the osteosynthesis. Although additional cerclage fixation of the distal patellar pole has been described in technical and clinical studies, its incremental biomechanical effect, in combination with locked plate fixation, was not specifically quantified in a model of a distal pole fracture with limited distal screw fixation.

The aim of this biomechanical study was to investigate whether the addition of a McLaughlin cerclage to fixed-angle locking plate osteosynthesis of distal patellar pole fractures provides superior construct stability compared to locked plating alone under conditions of limited distal screw placement. It was hypothesized that the combined use of a McLaughlin cerclage with plate osteosynthesis would result in significantly reduced interfragmentary displacement under cyclic loading compared with plating alone.

## Methods

### Specimen preparation

Sixteen unpaired fresh-frozen human cadaveric knees without prior bony or ligamentous injuries of the lower extremities were included in the study and randomly assigned by coin flip to two groups, with allocation constrained to obtain exactly eight specimens per group. One group underwent osteosynthesis with a locking plate alone (mean age 67.25 ± 11.5 years; 3 females, 5 males; 4 left, 4 right), while the second group received additional McLaughlin cerclage (mean age 67.75 ± 12.6 years; 4 females, 4 males; 6 left, 2 right). The experiments were performed with permissions from the responsible institutional ethics review board (Reference number 2022-195-f-S). The study was conducted in accordance with the applicable ethical principles of the Declaration of Helsinki. After thawing the specimens at 7 °C for 24 h, all surrounding cutaneous and subcutaneous tissue was removed. A standardized horizontal osteotomy was performed using an oscillating saw and chisel to simulate a distal patellar fracture involving the distal pole. The resulting fracture model retained approximately the distal third of the patella as a separate fragment.

This configuration was chosen to replicate a frequently observed morphology in AO/OTA 34-C3 type fractures. In a computed tomography (CT) based mapping analysis of multifragmentary patella fractures, Zhan et al. identified a predominant transverse fracture pattern in the distal third of the patella with associated comminution distal to the main fracture line [43]. Similarly, Misir et al. found inferior pole involvement in up to 93.2% of the C3 fractures [22].

**Fig. 1:**
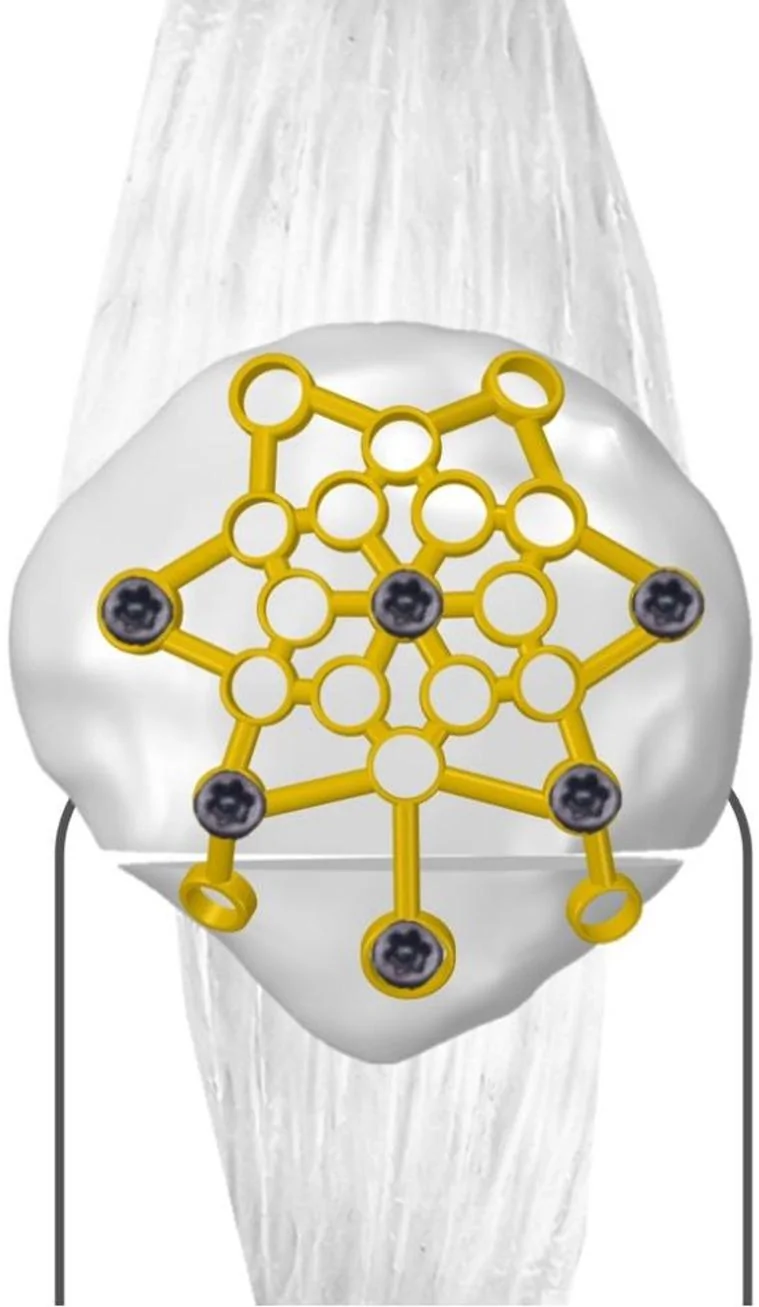
Schematic representation of patella with distal pole fracture. Plate osteosynthesis was performed with one screw in the distal pole fragment. Additional McLaughlin cerclage wiring was performed between the middle and distal third of the patella and the tibial tubercle

Fracture reduction and osteosynthesis were performed using a star-shaped precontoured plate (Variable Angle Locking Patella Plating System, DePuy Synthes, Zuchwil, Switzerland), applied to the anterior patellar surface according to the manufacturer’s technique guide. The demonstrated superiority of angular stable plates over other primary fixation methods in both biomechanical and clinical studies, along with their frequent use in fractures involving the distal pole, made them a suitable basis for hybrid fixation and an appropriate control group [5, 25]. In the distal fragment, only one of three possible plate screws was inserted to replicate the clinically typical scenario of small inferior pole fractures (see Fig. 1). In such cases, fragment size and limited bone stock often prevent full utilization of all screw holes, because of frequently small and multiple fragments of less than 1 cm³ in volume [22, 43, 45].

In the group receiving additional McLaughlin cerclage, two tunnels were drilled to accommodate the wire configuration. The first tunnel was positioned horizontally between the middle and distal third of the patella. The second tunnel was drilled through the tibial tubercle, according to the original technique described by McLaughlin for transosseous augmentation of the extensor mechanism [1, 21]. All reductions and fixation procedures were performed by the same surgeon (A.D.). Each 1.5-mm stainless-steel cerclage wire was manually tensioned and secured with four twists on each side. No standardized tensioning device was used, and wire pretension was not quantified. Patellar height and potential overconstraint of the extensor mechanism were assessed visually to avoid a visible change in patellar position.

### Biomechanical testing module

Biomechanical testing was conducted using a servo-hydraulic material testing machine (Zwick Roell, Germany) equipped with a 10 kN load cell (accuracy ± 0.03%). Specimens were mounted in a custom-made testing rig, allowing for horizontal fixation of the femur while leaving the tibia freely mobile. An additional weight of 2 kg was added to the tibia to replicate the physiological weight of the lower extremity. The design of the test setup was adapted from previously published biomechanical protocols investigating patellar fracture fixation constructs [31, 35, 37, 41].

**Fig. 2:**
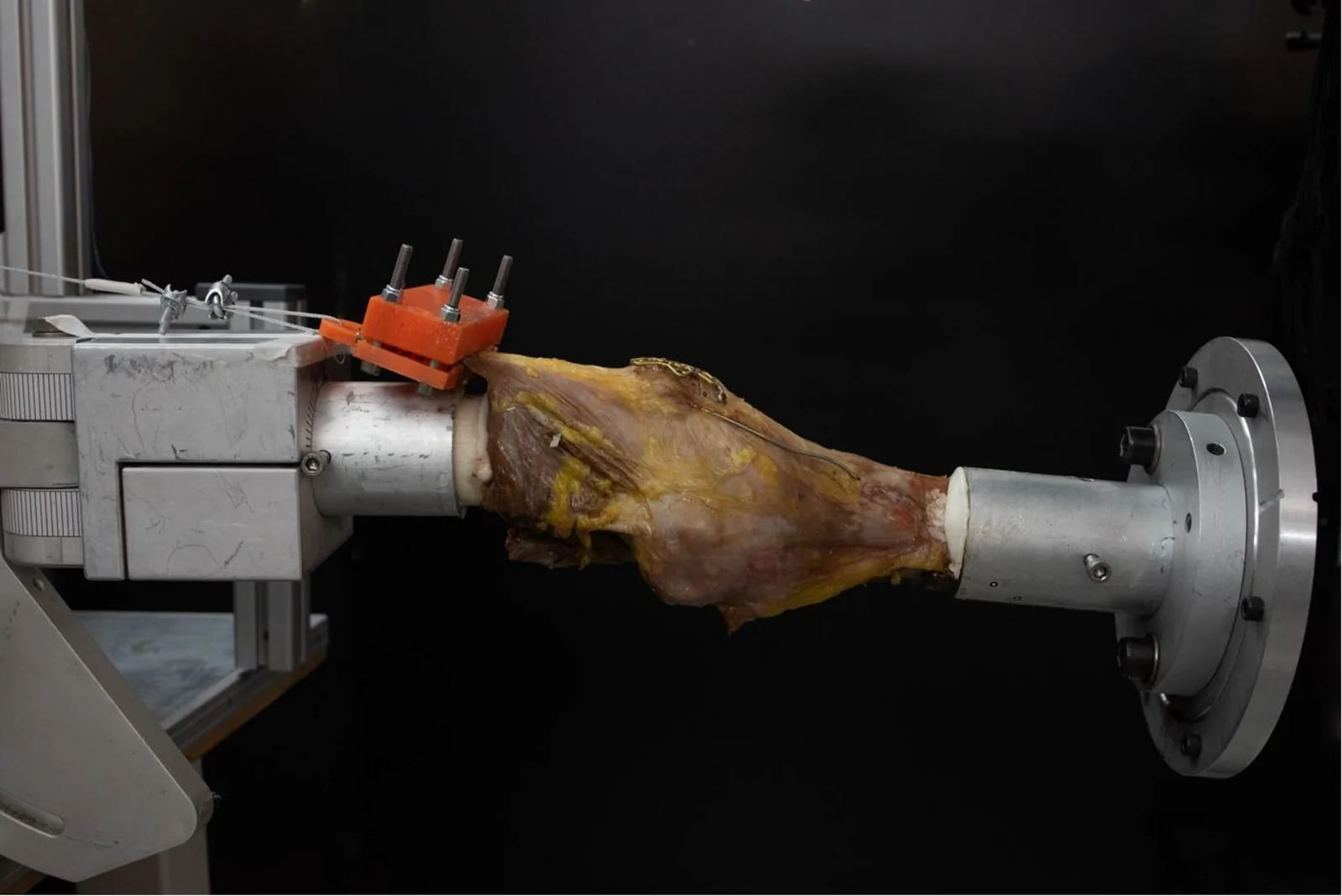
Experimental test setup. A tensile force is applied by a materials testing machine to the knee joint by attaching a wire cable to the quadriceps tendon, via a clamp, allowing the knee to be moved between flexion and extension. An additional weight of 2 kg was added to the tibia to replicate the physiological weight of the lower extremity

Physiological loading of the reconstructed extensor mechanism was simulated by redirecting axial displacement of the testing machine’s crosshead into tensile forces along the femoral axis using a system of deflected steel cables. The quadriceps tendon was carefully isolated from adjacent soft tissues and secured in a 3D-printed clamp specifically designed to prevent slippage. This clamp was connected to the steel cable system, allowing for controlled and reproducible transmission of tensile force (see Fig. 2).

Each specimen underwent cyclic loading consisting of 3000 cycles at a frequency of 0.1 Hz. The number of 3000 cycles was chosen to ensure a standardized, repetitive loading period sufficient for evaluating the progressive displacement of the structure, while the frequency of 0.1 Hz allowed for controlled knee movement and reliable optical tracking during each loading cycle. The protocol was initiated with the knee flexed to 90°, applying an initial preload of 30 N to the quadriceps tendon. From this position, the specimen was cyclically extended to simulate early postoperative motion. The maximum tensile force applied to the quadriceps tendon during each extension cycle reached 320 N, consistent with prior biomechanical patella fixation studies and within the range of reported physiological loading during early rehabilitation protocols [28]. Construct failure was identified as loss of fixation preventing continued cyclic loading. If failure occurred, testing was stopped and the last observed displacement was carried forward for subsequent cycles in the original analysis. These carried-forward values were not measurements obtained after failure. Failure timing was localized to the interval between the last valid stereooptical measurement and the next scheduled measurement. No subsequent load-to-failure testing was performed, and therefore no stiffness was evaluated.

### Digital image correlation (DIC)

Utilizing multiple cameras, DIC enables 3D motion tracking of fracture fragments. In this study, a commercial DIC system (ARAMIS, GOM GmbH, Braunschweig, Germany) was used to visualize and quantify the relative movements of the patellar fracture fragments.

**Fig. 3:**
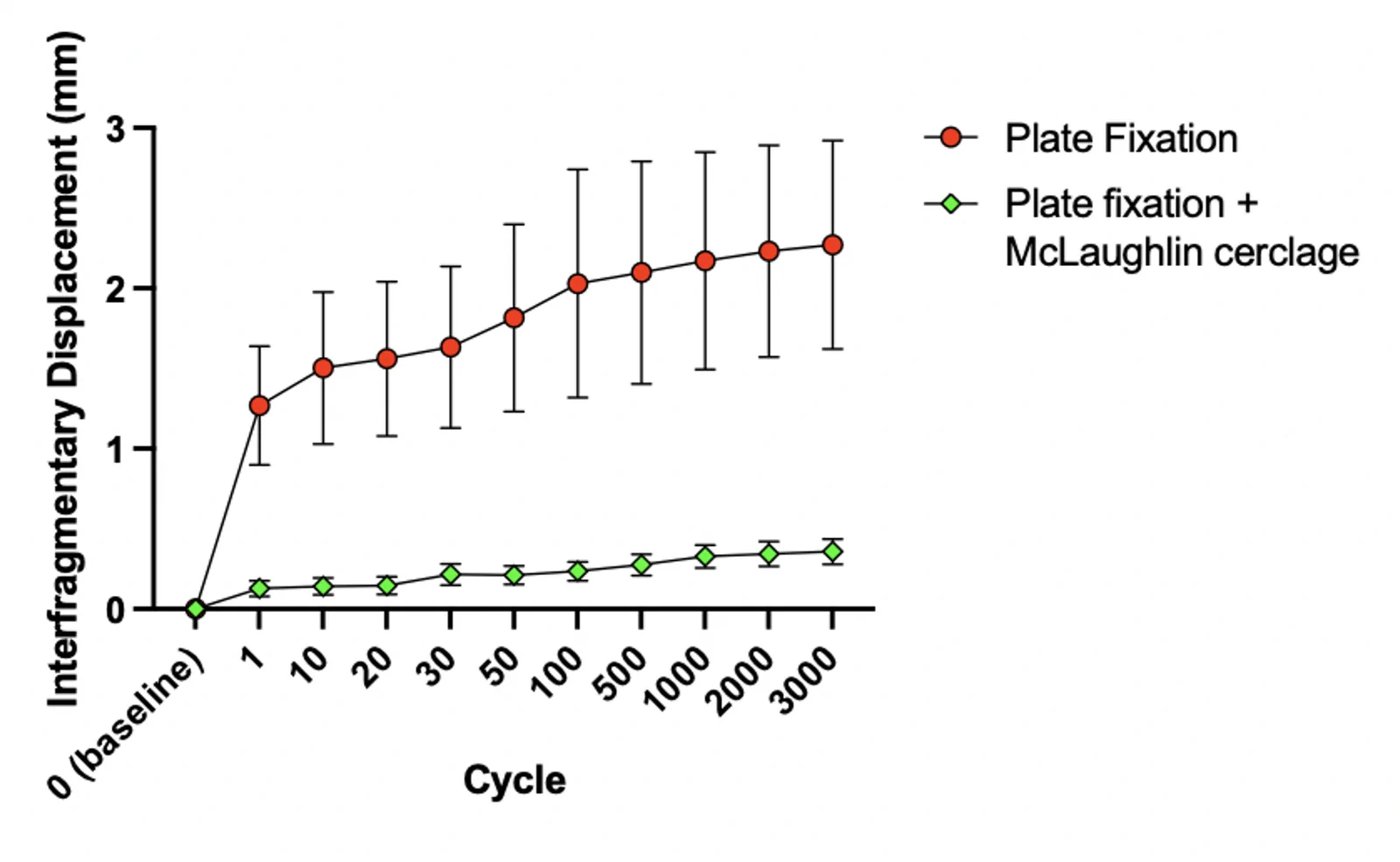
Interfragmentary displacement over loading cycles for plate fixation and additional McLaughlin cerclage, presented as mean ± standard error of the mean. Cycle 0 represents the unloaded zero-displacement reference

Optical markers with an inner diameter of 0.4 mm were applied, three at the proximal and two at the distal fragment. Marker positions were recorded first in the unloaded condition, and then at full load after 1, 10, 20, 30, 50, 100, 500, 1000, 2000, and 3000 cycles at 320 N of quadriceps pull force. The unloaded condition corresponded to cycle 0 and was defined as 0 mm displacement for all specimens. Cycle 0 is shown in Fig. 3 for visualization only and was not part of the inferential statistical analysis.

Image analysis was performed using GOM Correlate Pro (GOM GmbH, Braunschweig, Germany). Distances were calculated between the optical markers placed at the proximal and distal fracture fragments for each cycle. Changes in the distances were interpreted as a measure of interfragmentary displacement caused by the applied loading.

### Statistical analysis

A priori sample size calculation was performed using G*Power (version 3.1; University Düsseldorf, Düsseldorf, Germany) [11]. The primary endpoint was interfragmentary displacement after 3000 loading cycles. An expected standard deviation of 1.6 mm was based on the variability reported in a cyclic biomechanical study of patellar tendon repair [27]. With an assumed between-group difference of 3.0 mm, a two-sided independent-samples t test (Cohen’s d = 1.875; α = 0.05; power [1 − β] = 0.80; allocation ratio = 1:1) required six specimens per group (total *n* = 12; actual power = 0.833). Eight specimens were included per group to account for biological variability and potential specimen loss. The 3.0-mm assumption represented a large between-group contrast for sample-size planning, rather than a validated minimum clinically important difference.

Statistical analysis was performed using Prism (versions 10 and 11.1, GraphPad Software, San Diego, CA, USA). Normality of the data distribution was assessed using the Shapiro–Wilk test. Interfragmentary displacement measured from cycles 1 through 3000 was analyzed using a two-way repeated-measures analysis of variance (ANOVA), with fixation technique as the between-specimen factor, loading cycle as the within-specimen factor, and specimen as the repeated unit. Sphericity was not assumed. Therefore, Geisser–Greenhouse correction was applied. The fixation technique × loading cycle interaction was included in the model. At the primary endpoint of 3000 cycles, the groups were compared using a two-sided Welch’s unpaired t test. No multiplicity adjustment was applied because only this planned comparison was interpreted. A post hoc sensitivity analysis excluded the three plate-fixation constructs that failed during cyclic loading and repeated the 3000-cycle comparison using the same two-sided Welch’s unpaired t test. The overall level of significance was set at *p* < 0.05. Results are reported as mean ± standard deviation unless otherwise stated.

## Results

The two-way repeated-measures ANOVA demonstrated significant main effects of fixation technique (F = 8.32, *p* = 0.0120) and loading cycle (F = 4.52, *p* = 0.0496). The fixation technique × loading cycle interaction was not significant (F = 1.76, *p* = 0.2057). Three constructs in the plate-fixation group failed during cyclic loading due to pullout of the screw from the distal pole fragment. Failure occurred between cycles 10 and 20, 30 and 50, and 100 and 500, respectively. No construct in the additional McLaughlin cerclage group failed.

Interfragmentary displacement increased across the loading protocol in both groups and was lower in the additional McLaughlin cerclage group at every recorded cycle (Table 1; Fig. 3).

At the primary endpoint of 3000 cycles, displacement was 2.27 ± 1.84 mm for plate fixation and 0.36 ± 0.22 mm for additional McLaughlin cerclage, corresponding to an 84% lower mean displacement with additional McLaughlin cerclage (mean difference, 1.91 mm; 95% CI, 0.38–3.45 mm; *p* = 0.0215).

The sensitivity analysis excluding failed constructs did not alter the direction or statistical significance of the between-group difference at 3000 cycles. Displacement was 1.14 ± 0.19 mm in the five remaining plate-fixation constructs and 0.36 ± 0.22 mm in the eight constructs with additional McLaughlin cerclage (mean difference, 0.78 mm; 95% CI, 0.52–1.04 mm; *p* < 0.0001).

**Table 1:** Interfragmentary displacement at the distal pole of the patella, presented as mean ± standard deviation for each group across the loading cycles. Values at the individual cycles are descriptive; the only between-group comparison at a specific cycle was the prespecified comparison at cycle 3000

Cycle	Interfragmentary displacement (mm)	
	Plate fixation	Additive McLaughlin cerclage
1	1.27 ± 1.04	0.13 ± 0.14
10	1.50 ± 1.34	0.14 ± 0.15
20	1.56 ± 1.36	0.15 ± 0.15
30	1.63 ± 1.42	0.22 ± 0.19
50	1.82 ± 1.65	0.21 ± 0.16
100	2.03 ± 2.01	0.24 ± 0.17
500	2.10 ± 1.96	0.28 ± 0.19
1000	2.17 ± 1.91	0.33 ± 0.20
2000	2.23 ± 1.87	0.34 ± 0.22
3000	2.27 ± 1.84	0.36 ± 0.22

## Discussion

The most important finding of this study was that the addition of a McLaughlin cerclage to fixed-angle plate osteosynthesis of the patella significantly reduced interfragmentary displacement, thereby increasing biomechanical stability compared to plating alone in the treatment of distal patellar pole fractures. This finding was retained in the sensitivity analysis excluding failed constructs, although the estimated between-group difference was smaller.

Previous biomechanical studies demonstrated the superiority of fixed-angle locked plating over TBW or lag screw constructs in complex patellar fracture models [38]. A biomechanical study on human cadaveric knee specimens reported significantly reduced interfragmentary displacement with variable-angle locked plating compared to TBW in AO/OTA 34-C3 fractures [32]. Similarly, another biomechanical study confirmed that locked plating withstands higher cyclic loads with less displacement than TBW [41]. However, all these studies investigated optimal fixation scenarios with two or more screws per fragment, which is not necessarily representative of clinical reality. The study by Stoffel et al. [30] addressed this common comminuted fracture scenario by using a single distal pole screw and comparing bicortical screw fixation with angle-stable fixation. Indeed, failure rates of up to 40% are reported for multifragmentary fractures treated with plating alone [15]. Limited evidence exists regarding the possible advantages of hybrid fixation techniques. A clinical study on thirty patients demonstrated high union rates (96%) using locking plates combined with tension band wiring using two parallel K-Wires and an anterior figure-of-eight wire [6]. A recent meta-analysis supports the notion that supplementary fixation reduces bone union-related complications in inferior pole fractures [17]. Although the literature on the McLaughlin cerclage is sparse, Ahrberg et al. reported favorable outcomes using this technique for distal patellar fractures [1], and Ellwein et al. observed good functional results when combining locked plating with a McLaughlin cerclage [10]. Recent clinical studies have yielded conflicting results regarding the need for additional fixation in cases of comminuted patellar fractures. Hoskins et al. observed a fixation failure rate of 40% with isolated fixation using a variable-angle plate, compared with 11.1% when additional augmentation was used, suggesting a possible protective effect of the additional fixation [15]. In contrast, Dadpey et al. [8] reported a low overall failure rate of 2.9%, with no failures occurring in the group receiving plate fixation alone; however, the authors emphasized that the study was not sufficiently powered to determine whether augmentation offers additional clinical benefit. It is noteworthy that the only fixation failure reported by Dadpey et al. occurred in an AO/OTA 34-C3 fracture involving the distal pole. Taken together, these results suggest that the clinical necessity of routine augmentation remains unclear and may depend, in particular, on the fracture morphology and the stability of the distal pole fixation.

Experimental fracture-healing research has discussed an absolute gap of approximately 2 mm [8]. Larger interfragmentary gaps have been associated with impaired neovascularization and healing in experimental models [7, 44]. In the the present study, mean displacement exceeded 2 mm with plate fixation alone and remained below 2 mm with augmentation. After exclusion of failed constructs during sensitivity analysis, both group means were below 2 mm, while the displacement difference remained statistically significant. The findings therefore support reduced displacement under the tested conditions when cerclage augmentation is performed. The relevance of this topic is supported by a survey conducted among experienced trauma surgeons, which found that, regardless of the type of osteosynthesis, most surgeons used cerclage augmentation for comminuted patellar fractures [13]. The present study therefore provides new insights and underlines that especially in fractures involving the distal patellar pole, an additional McLaughlin cerclage might be of advantage as fracture reduction may be better maintained by adding an additional cerclage. Moreover, this finding is consistent with biomechanical studies showing improved construct stability when patellar tendon repair, which is biomechanically comparable to fixation of a distal pole avulsion, is augmented with a McLaughlin cerclage [26, 27]. However, the present study does not allow conclusions to be drawn regarding the relative biomechanical efficacy of alternative augmentation techniques, such as additional screw fixation, suture-based constructs, or other supplementary fixation methods.

This study has several strengths and limitations. It addresses a clinically relevant question and focuses on a specific technical aspect of fixation, allowing a targeted comparison of two fixation strategies using a reproducible biomechanical methodology designed to replicate physiological patellar loading and motion as closely as possible. Furthermore, interfragmentary displacement was measured using a highly precise optical measurement system, enabling objective, high-resolution quantification during repetitive loading.

However, cadaveric models cannot replicate biological healing, bone remodeling, or neuromuscular control and cannot assess clinical outcomes or complications such as implant irritation, patella baja, postoperative stiffness, or the need for implant removal. These factors limit direct clinical applicability. Furthermore, no specific bone-density measurements were available for the body donors. Although age may serve as an indicator, the lack of direct assessment limits generalizability. Although the standardized fracture model was based on CT-derived morphology of distal pole fractures, it represents a simplified configuration compared with the variability and comminution encountered clinically. This simplification was necessary to ensure biomechanical reproducibility and reduce interspecimen variability. Manual cerclage tensioning without a standardized tensioning device or quantified pretension may have introduced variability. No load-to-failure testing was performed, and construct stiffness was not evaluated. Therefore, the conclusions are limited to interfragmentary displacement during cyclic loading. The cyclic-loading protocol was designed to approximate early postoperative activity but cannot fully reproduce the complex multidirectional forces acting on the patella during rehabilitation.

## Conclusion

An additional McLaughlin cerclage reduced interfragmentary displacement in distal patellar pole fractures. In cases of unstable distal pole patella fractures, the additional use of a McLaughlin cerclage may therefore be considered to enhance construct stability.

## Data Availability

No datasets were generated or analysed during the current study.
